# circPTP4A2 knockdown suppresses NSCLC progression via regulating proliferation and activating anti-tumor immunity

**DOI:** 10.1186/s13019-024-02964-9

**Published:** 2024-07-16

**Authors:** Chun Wang, Bin Xu, Chengzhi Tao, Huan Lin, Dan Liu, Haitao Zhang

**Affiliations:** Department of Respiratory Medicine, Nanjing Chest Hospital, Affiliated Nanjing Brain Hospital, Nanjing Medical University, 215 Guangzhou Road, Nanjing City, Jiangsu Province 210029 China

**Keywords:** Hsa_circ_0007364, Immune, NSCLC, Diagnosis, Progression

## Abstract

**Background:**

With a considerable variety of cancer subtypes, Non-small cell lung cancer (NSCLC) poses a substantial threat to public health, affecting a large number of individuals and resulting in a high mortality rate. Circular RNA (circRNA) has been applied in various diseases, including cancers. This study aims to investigate the clinial significance and functional role of circPTP4A2 in NSCLC.

**Methods:**

The serum and tissue samples were collected for detecting circPTP4A2 expression in NSCLC using reverse transcription-quantitative polymerase chain reaction (RT-qPCR). Actinomycin D was used to treat NSCLC cells to detect circPTP4A2 stability. The CCK-8 and Transwell assays were utilized to assess the effects of circPTP4A2 in NSCLC cells. The ELISA assay and cytotoxicity analysis were used to detect the roles of circPTP4A2 in immune escape.

**Results:**

The serum and tissue circPTP4A2 expression was upregulated in NSCLC. The high circPTP4A2 had a relatively high value in differentiating NSCLC patients from healthy individuals. The proliferation, invasion, and immune escape were repressed by circPTP4A2 knockdown.

**Conclusions:**

High circPTP4A2 has the potential to be a diagnostic biomarker in NSCLC. Silencing of circPTP4A2 receded the progression of NSCLC and enhanced antitumor immunity, which might provide potential targets and new ideas for improving the diagnosis and effect of immunotherapy in NSCLC patients.

**Supplementary Information:**

The online version contains supplementary material available at 10.1186/s13019-024-02964-9.

## Highlights

Increased circPTP4A2 has potential diagnostic value in NSCLC patients.

Silencing of circPTP4A2 inhibits NSCLC cellular activities.

circPTP4A2 knockdown increased NSCLC antitumor immune response.

**Supplementary Information:**

The online version contains supplementary material available at 10.1186/s13019-024-02964-9.

## Background

Non-small cell lung cancer (NSCLC) accounts for approximately the majority of lung cancer cases, which is still one of the most common cancers worldwide [1, 2]. Primary NSCLC cells have a preference to spread to lymph nodes, contralateral lung, and distant organs such as bone, brain, and liver, which usually have a low response to initial antitumor therapy and a high frequency of recurrence after treatment [2]. The problem that has been plaguing the clinic is that the survival rate of NSCLC patients with metastasis is still low and with poor prognosis [3]. Once metastasis occurs, it not only means loss of surgical opportunity but also indicates poor prognosis. In recent years, although novel targets and therapies like targeted therapy and immunotherapy have exhibited promising effects in extending the survival of patients with NSCLC, the outcome for those with metastatic NSCLC is still not satisfactory [4, 5]. Early diagnosis and early treatment will increase the survival rate of patients. When NSCLC is found to be metastasized, it is necessary to identify the molecules that facilitate its metastasis and give targeted treatment.

Circular RNAs (circRNAs) are a newly discovered class of nonlinear single-stranded non-coding RNAs that widely exist in the human body [6, 7]. Their distinctive covalently closed loop structure makes them resistant to degradation by exonucleases and debranching enzymes, resulting their robust biological stability and tissue-specificity characteristics, which make them potential biomarkers in diseases [6]. The development of NSCLC involves many molecular abnormalities and is accompanied by complex biological processes [8]. The expression of non-coding RNAs is a useful indicator to describe the molecular characteristics of NSCLC and plays a crucial role in the regulation of the immune system [9, 10]. Many circRNAs function as oncogenic or inhibitory non-coding RNAs that participate in tumor progression and tumor immunity of NSCLC, such as circC190 and circNDUFB2 [11, 12]. Hsa_circ_0007364 (circPTP4A2, located on chr1:32381495–32,385,259) is upregulated in cervical cancer and promotes tumor cell progression through regulating miR-101-5p/MAT2A axis [13]. In addition, abnormal expression of circPTP4A2 was involved in many diseases, such as cerebral ischemic stroke and intrauterine adhesions [14–16]. However, whether circPTP4A2 has a functional role in NSCLC remains unclear.

Based on the GEO database (GSE158695), we explored the expression pattern of circPTP4A2 in NSCLC and probed its clinical diagnostic performance. The circPTP4A2 knockdown displayed inhibitory effects on NSCLC cellular activities and immunity, aiming to provide potential targets and new ideas for improving the diagnosis and effect of immunotherapy in NSCLC patients.

## Methods

### Sample information

The paired NSCLC tissues and adjacent tissues that underwent surgery in Nanjing Chest Hospital, Affiliated Nanjing Brain Hospital, Nanjing Medical University from May 2022 to July 2023 were collected. All patients (*n* = 122) were newly diagnosed with NSCLC by pathologies. The patients’ age ranges from 25 to 76 years old (51.81 ± 10.35 years). The collected tissue samples were placed in RNase-free tubes and transferred in liquid nitrogen for long-term storage. The fasting blood samples (5 ml) were obtained from patients with NSCLC in the morning after an overnight fast. Besides, fasting blood samples were collected from 100 healthy adults (ranging from 25 to 70 years, 48.76 ± 13.84 years) during routine physical examination as control. The serum samples were separated within 4 h after fasting blood samples collection and stored at -80 °C. Clinical characteristics of NSCLC patients were collected and recorded. All participants signed informed consent before specimen collection and this study was approved by the Ethics Committee of Nanjing Chest Hospital, Affiliated Nanjing Brain Hospital, Nanjing Medical University.

### Cell culture and transfection

Human bronchial epithelial cell line BEAS-2B and NSCLC cell lines (H1650, Calu-3, H1299, A549) were purchased from Procell (China) and cultured in RPMI 1640 (Hyclone, USA) that added 10%FBS (Gibco) at 37 °C in a humidified atmosphere with 5% CO_2_.

circPTP4A2 small interfering RNA (siRNA) and scrambled siRNA NC were purchased from Sangon Biotech (Shanghai, China). The NSCLC cells in the logarithmic growth phase were digested, centrifuged, re-suspended, counted, inoculated into 6-well plates at a cell density of 2 × 10^5^ cells/well, and cultured overnight in an incubator. Then transfection was achieved with the help of a Lipofectamine 2000 transfection kit (Invitrogen).

### RNA extraction and reverse transcription-quantitative polymerase chain reaction (RT-qPCR)

Cancer tissue or adjacent tissue samples were weighed and ground on dry ice then added into the centrifuge tube and added TRizol to extract total RNA. Total RNA from serum samples and cells was extracted using TRIzol (Sigma-Aldrich). After purity and quality measurement, total RNA was reverse transfected into cDNA using the Takara reverse transcription kit PrimeScript™ RT reagent Kit with gDNA Eraser. The PCR amplification was performed using a TB Green PCR Master Mix kit (Takara). The circPTP4A2 and mRNA levels were calculated by the 2^−ΔΔCt^ method and normalized by β-actin. The sequences used in the experiments were listed in Supplementary Table 1.

### Actinomycin D assays

NSCLC cells were exposed to actinomycin D (2 µg/ml; Sigma, USA) in 6-well plates for indicated time points. The RT-qPCR was used to analyze the RNA stability.

### Cell functional CCK-8 experiments

Cell Counting kit-8 (CCK-8) proliferation assay: NSCLC cells were adjusted to 4000 cells/well and inoculated into 96-well plates for 24 h at 37 °C. The CCK-8 (10 µl; Dojindo, Japan) was added to the wells and continued to culture for another 1 h. The absorbance of cells (450 nm) was measured to reflect cell proliferation abilities.

### Transwell assays

Twenty-four well chambers with or without Matrigel (Corning) were used to measure cell motility, including migration potential and invasive capacities. Transfected NSCLC cells resuspended in serum-free culture medium (5 × 10^4^ cells) were seeded in the upper chamber of the Transwell chamber in the absence of serum in the medium. Using a pipetting gun, a complete culture medium was carefully added to the bottom of the transwell chamber, and FBS was used as an attractant. After 48 h of culture, transwell chambers were washed twice using PBS solution. Cells on the upper layer of the transwell chamber were gently removed using a moist cotton swab, and then cells on the lower surface of the membrane were stained and counted.

### Cytotoxicity analysis

Peripheral venous blood from healthy adults was collected in anticoagulated tubes. Then Ficoll gradient centrifugation was used to isolate peripheral blood mononuclear cells (PBMCs). Some PBMC cells were resuspended at a density 1 × 10^6^ cells/ml in RPMI 1640, added with 1000 U/ml IFN-γ, 300 U/ml recombinant human IL-2, 10% FBS (Invitrogen), and 25 mmol/L HEPES (Sigma-Aldrich) after 24 h (supplement 100 µg/L anti-CD3 and 100 U/ml IL-1α). Cytokine-induced killer (CIK) cells were harvested after 13 days of culture.

NSCLC cells as targeted cells were cultured with CIK cells as effector cells (effector: target cells ratio 15:1) in 96-well plates and maintained for 24 h. Then CCK-8 assay was used to measure the cell cytotoxicity by detecting the absorbance at 450 nm and the survival rate was calculated.

### Enzyme-linked immunosorbent assay (ELISA)

Transfected NSCLC cells were co-cultured with PBMC cells (treated with 5µg/ml PHA; 5 × 10^5^ PBMC cells). The levels of interferon-γ (IFN-γ) and tumor necrosis factor-alpha (TNF-α) in the cell culture medium were detected by a human IFN-γ ELISA kit (Elabscience, Jiangsu, China) and a human TNF-α ELISA kit (Elabscience).

### Statistical analysis

Data are presented as mean ± standard deviation (SD) or case numbers (n), respectively. For continuous variables, the student’s t-test (normally distributed data) was used to compare between two groups. One-way analysis of variance (ANOVA) (three or more groups) was performed followed by an appropriate post hoc test. The receiver operator characteristic (ROC) curve was conducted to evaluate the diagnostic value of circPTP4A2. All statistical tests were performed using SPSS 26.0 Software and GraphPad Prism 9.0 and a *P* value less than 0.05 (two-sided) was considered statistically significant.

## Results

### circPTP4A2 expression detection and validation in NSCLC

**Fig. 1:**
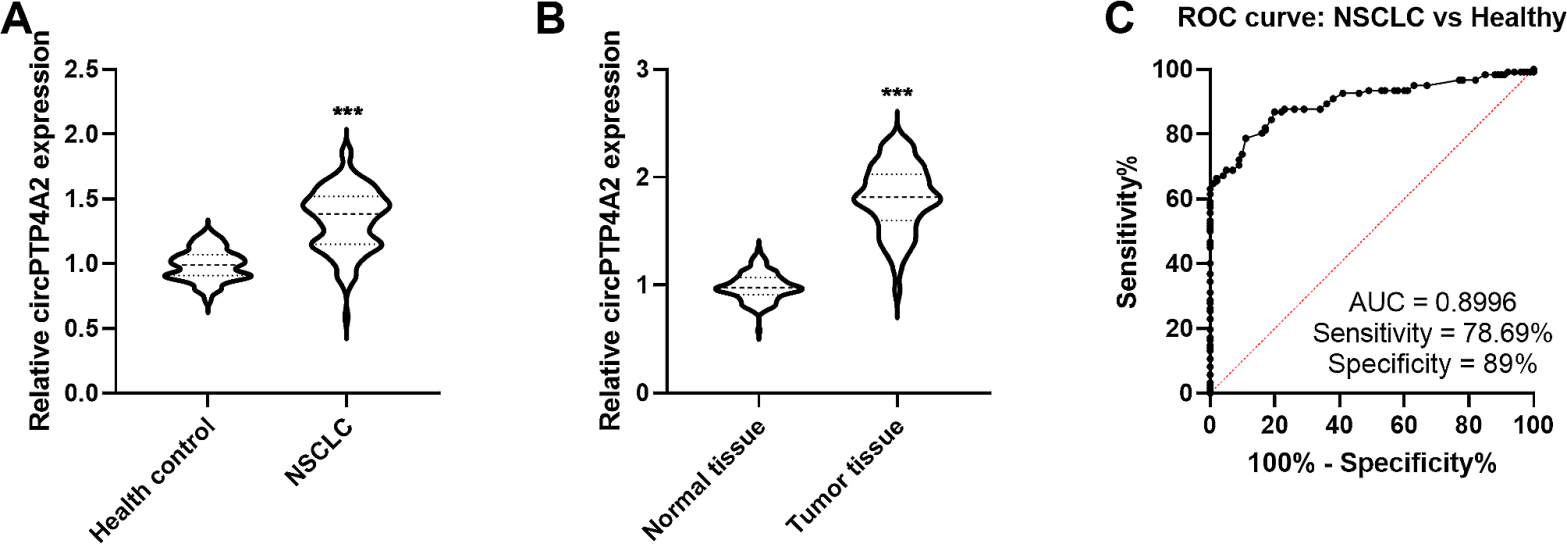
circPTP4A2 expression and its diagnostic value in NSCLC. (**A**) Serum circPTP4A2 levels in NSCLC patients and health control. (**B**) circPTP4A2 expression was validated in NSCLC tissues. (**C**) ROC curve was conducted to evaluate the diagnostic value of circPTP4A2. ***P < 0.001

The differentially expressed circRNAs in NSCLC was downloaded from GSE158695 with the GEO2R tool. The volcano plot was constructed with the criteria of log|FC| > 2 and *P* value < 0.05 that displayed 12 upregulated circRNAs and 72 downregulated circRNAs (Supplementary Fig. 1A). The expression trend of these 84 circRNAs were shown in heatmap (Supplementary Fig. 1B). Based on literature and the upregulated circRNAs, the upregulated circ_0007364 (circPTP4A2) was selected for validation. Firstly, the serum circPTP4A2 expression was measured in NSCLC patients and healthy controls using RT-qPCR. Compared with healthy control, the levels of serum circPTP4A2 were higher in NSCLC patients (Fig. 1A). Then circPTP4A2 expression was validated in tissue specimens and the data indicated that its expression was increased in tumor tissues in contrast to adjacent normal tissues (Fig. 1B). Based on its specific expression pattern in NSCLC patients, its diagnostic performance was probed. ROC curve analysis showed that serum circPTP4A2 had a relatively high AUC value to distinguish NSCLC patients from healthy control (Fig. 1C, AUC = 0.8996, *P* < 0.001).

### Correlation between circPTP4A2 and clinical parameters of NSCLC patients

According to the mean expression of serum/tissue circPTP4A2 in NSCLC patients, the patients were divided into two groups, high circPTP4A2 expression and low circPTP4A2 expression. Basic clinical information of patients was collected in Table 1. We observed that patients with big tumor size, poor differentiation, lymph node metastasis, and high TNM stage have high serum circPTP4A2 expression. Whereas other basic clinical characters showed no significant correlation with serum circPTP4A2. Besides, positive lymph node metastasis and advanced TNM stages were correlated with high tissues circPTP4A2.

**Table 1:** Correlation between circPTP4A2 expression and basic clinical information of NSCLC patients

Factors	Cases	Serum circPTP4A2 expression		*P*-value	Tissue circPTP4A2 expression		*P*-value
	(*n* = 122)	High (*n* = 65)	Low (*n* = 57)		High (*n* = 64)	Low (*n* = 58)	
Sex				0.057			0.081
Female	51	22	29		22	29	
Male	71	43	28		42	29	
Age				0.461			0.580
< 50 years	62	31	31		31	31	
≥ 50 years	60	34	26		33	27	
Tumor size				0.041			0.064
< 5 cm	65	29	36		29	36	
≥ 5 cm	57	36	21		35	22	
Smoking history				0.108			0.079
No	72	34	38		33	39	
Yes	50	31	19		31	19	
Differentiation				0.032			0.453
High-moderate	80	37	43		40	40	
Poor	42	28	14		24	18	
Lymph node metastasis				0.024			0.018
Negative	84	39	45		38	46	
Positive	38	26	12		26	12	
TNM stage				0.003			< 0.001
I-II	77	33	44		30	47	
III	45	32	13		34	11	

### circPTP4A2 expression and its functional effects in NSCLC cells

**Fig. 2:**
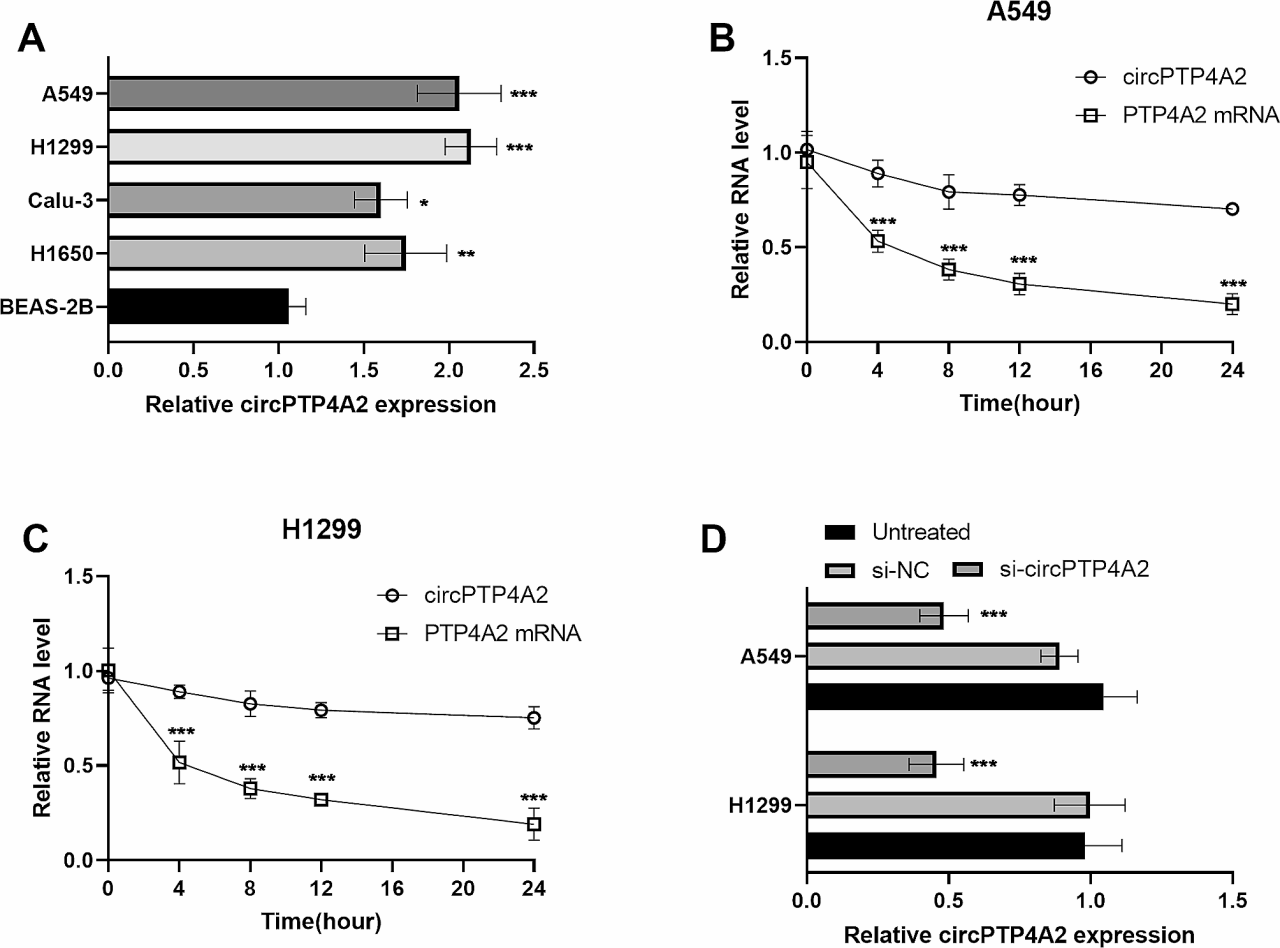
circPTP4A2 in NSCLC cells and transfection efficiency. (**A**) circPTP4A2 levels were higher in NSCLC cells than that in normal BEAS-2B cells. (**B**) and (**C**) After Actinomycin D treatment, the RNA abundance of circPTP4A2 and PTP4A2 was analyzed. (**D**) The levels of circPTP4A2 were measured after transfection. *P < 0.05, **P < 0.01, ***P < 0.001

The levels of circPTP4A2 in NSCLC cells were further explored. Compared with normal BEAS-2B cells, circPTP4A2 expression in these NSCLC cell lines was significantly upregulated (Fig. 2A). H1299 and A549 cells were treated with the transcription inhibitor actinomycin D, and then the circPTP4A2 and PTP4A2 mRNA expression in NSCLC cells were measured at different time points. The data show that circPTP4A2 is more stable than PTP4A2 mRNA (Fig. 2B and C). Then, Transfection using circPTP4A2 siRNA successfully decreased the circPTP4A2 expression in both A549 and H1299 cells (Fig. 2D).

**Fig. 3:**
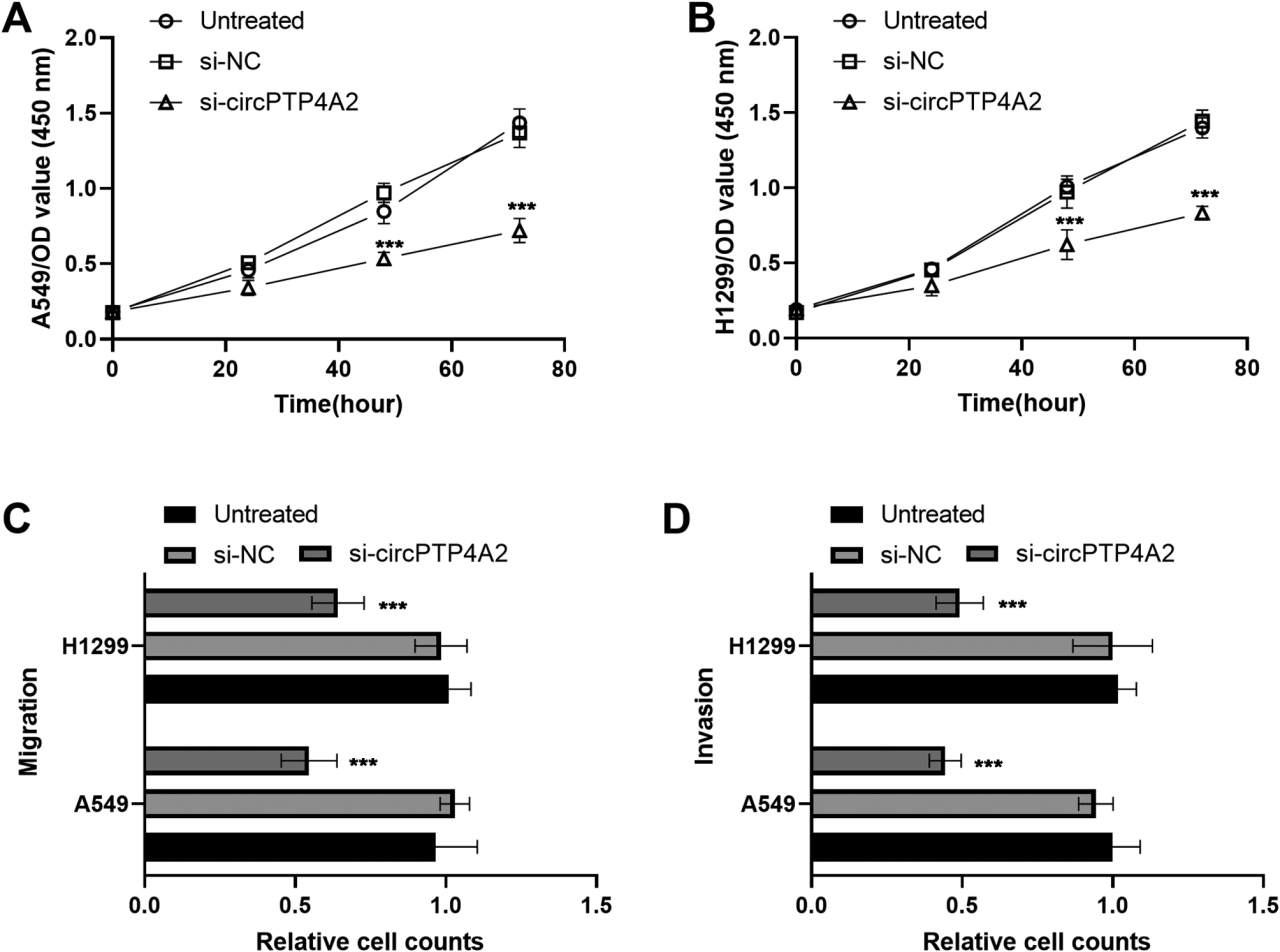
Influence of circPTP4A2 on cellular activities of NSCLC cells. (**A**) and (**B**). Silencing of circPTP4A2 decreased cell proliferation abilities in both A549 and H1299 cells. (**C**) and (**D**). The migration capacities and invasive potential of NSCLC cells were inhibited by si-circPTP4A2. ***P < 0.001

The functional experiments demonstrated that the proliferative capacities, migratory potential, and invasive abilities were repressed by circPTP4A2 knockdown (Fig. 3A and D).

### Silencing of circPTP4A2 inhibited immune escaper of NSCLC cells

**Fig. 4:**
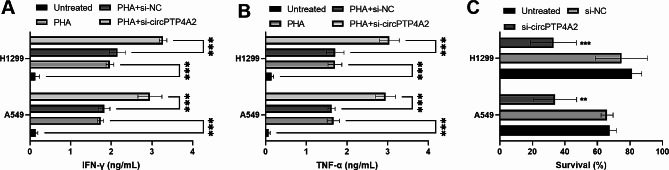
Decreased circPTP4A2 increased antitumor immune response. (**A**) and (**B**). ELISA assay was used to measure IFN-γ and TNF‐α levels in PHA-induced PBMCs cocultured with transfected NSCLC cells. (**C**) The CCK-8 assay was used to assess the survival rate of NSCLC cells (effector: target = 15: 1). **P < 0.01, ***P < 0.001

Considering the importance of immunotherapy, the effect of circPTP4A2 on antitumor immune response was explored. After co-culture PHA-stimulated PBMCs and transfected NSCLC cells, IFN-γ and TNF‐α levels were elevated in PHA-stimulated PBMCs, and interference of circPTP4A2 further enhanced IFN‐γ and TNF‐α levels (Fig. 4A and B). Using the effector-target cell ratio (15:1), the NSCLC cell survival rate was decreased by si-circPTP4A2 (Fig. 4C). The cytotoxic activity of CIK cells against si-circPTP4A2-transfected NSCLC cells was higher than that of untreated cells.

## Discussion

This part of the experiment is to explore the correlation between circPTP4A2 expression and NSCLC. The levels of circPTP4A2 in serum samples and tissues of patients with NSCLC were elevated. Moreover, its expression in NSCLC cells was higher than that in normal lung epithelial cells. Therefore, circPTP4A2 may be used as NSCLC-specific circRNA, and the detailed role of circPTP4A2 in NSCLC was further studied. Different pathological Subtypes of lung cancer showed diverse treatment responses [17, 18]. In recent years, NSCLC is becoming increasing susceptible to treatment with immunotherapeutic approaches, with certain patient subsets anticipated to repond favorably to this treatment modality, irrespective of their subtype of NSCLC [19, 20].

CircRNA was once considered to be a product of gene misplacing, due to the rapid sequencing and bioinformatics, more and more research has shown that circRNAs are widely present in organisms and are abundant [21]. In the GEO database (GSE158695) using three pairs of NSCLC tissues and corresponding non-cancerous tissues, circPTP4A2 was one of the upregulated circRNA in NSCLC. In the present data, serum circPTP4A2 showed a high expression level in NSCLC patients compared with healthy control. Then, high circPTP4A2 expression was validated in NSCLC tissues, which was consistent with (even higher) the pattern of the results in the serum of NSCLC patients. The circPTP4A2 expression in NSCLC cells was also higher than that in normal lung cells. Further clinical correlation analysis indicated that high circPTP4A2 expression was positively related to several crucial basic clinical parameters of NSCLC patients, especially positive lymph node metastasis and high TNM stages. These data suggest the potential oncogenic effect of circPTP4A2 in NSCLC.

Actinomycin D treatment assay displayed that the circPTP4A2 was more stable than linear PTP4A2 mRNA. The stable structure of circRNAs makes them possible to be biomarkers in diseases [22]. ROC curve displayed the serum circPTP4A2 has the potential to be a diagnostic biomarker in NSCLC. Besides, circPTP4A2 was indicated to be a diagnostic biomarker in acute ischemic stroke [23]. Similarly, circ_0070659 predicts poor prognosis of NSCLC patients and participates in tumor progression [24].

The specific expression and functional influence of circRNAs in cancer have been explored increasingly. For instance, circ_103615 and circPTP4A2 act as oncogenic circRNA or suppressor in NSCLC and modulate tumor cisplation resistance, and metastasis [25, 26]. The aberrant expression circPTP4A2 is involved in several diseases [14, 27]. For instance, increased circPTP4A2 contributes survival of human umbilical cord mesenchymal stem cells on SF-SIS scaffolds and endometrial repair [16]. In addition, circPTP4A2 contributed to cervical cancer tumorigenesis and might be a therapeutic target for treating cervical cancer [13]. Here, after silencing circPTP4A2 by si-circPTP4A2, the NSCLC cell growth abilities, migration, and invasion capacities were receded, revealing the inhibitory role of circPTP4A2 knockdown in NSCLC.

CircPTP4A2 was an immune-related circRNA that was increased and could improve sensitivity for the diagnosis of acute ischemic stroke [23]. Thus, we further probed the relationship between circPTP4A2 and immune escape. CircPTP4A2 knockdown further increased immune factor IFN-γ and TNF‐α levels but decreased the NSCLC survival after co-culture of NSCLC cells (targeted cells) and CIK cells (effector cells). These data suggest that downregulation of circPTP4A2 increased antitumor immune response.

However, there are several shortcomings in the current study. First, the sample size is limited and the clinical significance of circPTP4A2 needs to be validated in a big cohort. In addition, the further mechanism of circPTP4A2 in NSCLC needs to be investigated in the future.

## Conclusions

In summary, highly expressed circPTP4A2 in NSCLC has the potential to be a diagnostic biomarker in NSCLC. CircPTP4A2 accelerates NSCLC cellular capacities and immune escape in vitro. CircPTP4A2 has the potential biological function as a diagnostic biomarker and therapeutic target for NSCLC, which is expected to provide new ideas for the clinical treatment of NSCLC.

Supplementary Fig. 1 Differentially expressed circRNAs from GSE158695 dataset. (A) The volcano plot for the circRNAs data in NSCLC from GSE158695. (B) The heatmap for the upregulated and downregulated circRNAs in NSCLC from GSE158695.

### Electronic supplementary material

Below is the link to the electronic supplementary material.

Supplementary Material 1

Supplementary Material 2

## Data Availability

No datasets were generated or analysed during the current study.

## Abbreviations

circRNA: Circular RNA
NSCLC: Non-Small Cell Lung Cancer
PBMCs: Peripheral Blood Mononuclear Cells
siRNA: Small interfering RNA
RT-qPCR: Reverse transcription-quantitative polymerase chain reaction
CCK-8: Cell Counting kit-8
CIK: Cytokine-induced killer
ELISA: Enzyme-linked immunosorbent assay
IFN-γ: Interferon-γ
TNF-α: Tumor necrosis factor-alpha
ROC: Receiver operator characteristic
