# Supplementary figures and images for: circPTP4A2 knockdown suppresses NSCLC progression via regulating proliferation and activating anti-tumor immunity
Source: J Cardiothorac Surg. 2024 Jul 16;19:453. doi: 10.1186/s13019-024-02964-9 (PMC11250973; doi:10.1186/s13019-024-02964-9)

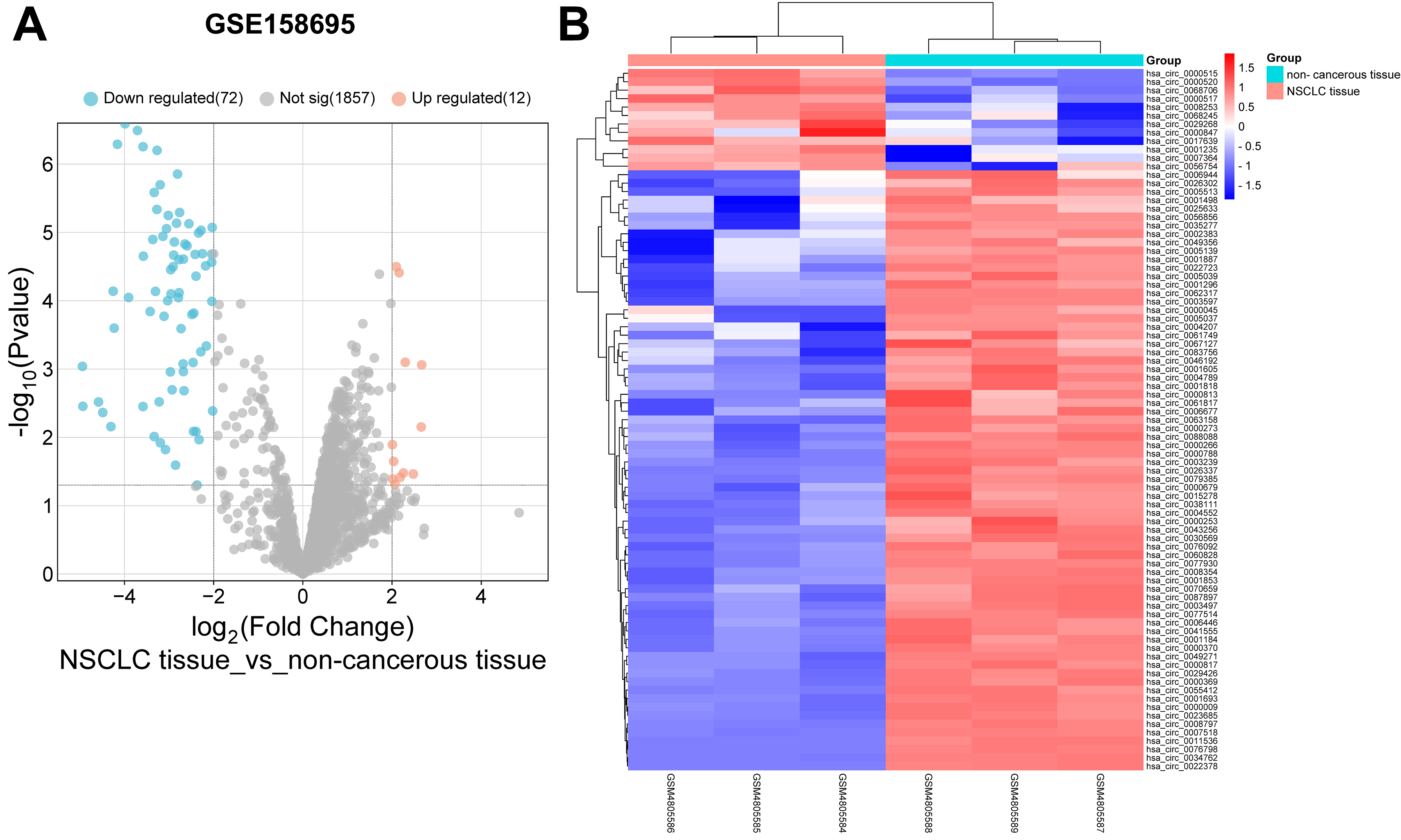

Supplement: Supplementary file 1 — Supplementary Material 1 [file 13019_2024_2964_MOESM1_ESM.tif]
