# Supplementary material for: circPTP4A2 knockdown suppresses NSCLC progression via regulating proliferation and activating anti-tumor immunity
Source: J Cardiothorac Surg. 2024 Jul 16;19:453. doi: 10.1186/s13019-024-02964-9 (PMC11250973; doi:10.1186/s13019-024-02964-9)
Supplement: Supplementary file 2 — Supplementary Material 2 [file 13019_2024_2964_MOESM2_ESM.docx]

**Supplementary table 1** Primers used in this study

| Name |  | Sequence (5'-3') |
| --- | --- | --- |
| CircPTP4A2 for RT-qPCR | forward | GGAGTGACGACTTTGGTTCG |
|  | reverse | TGTCAGCGAAAATGCTGTGC |
| PTP4A2 | forward | AGCCAGGTTGCTGTGTTGCAG |
|  | reverse | CACAGCAATGCCCATTGGTA |
| β-actin | forward | GTGACGTGGACATCCGTAAA |
|  | reverse | CAGGGCAGTAATCTCCTTCTG |
| CircPTP4A2 siRNA |  | ATCCACGTTCTAGTTTTTCGT |
| siRNA NC |  | UUCUCCGAACGUGUCACGUTT |
